# Prognostic Efficacy of Tumor-Stroma Ratio in Women With Breast Cancer: A Meta-Analysis of Cohort Studies

**DOI:** 10.3389/fonc.2021.731409

**Published:** 2021-12-16

**Authors:** Pengli Jiang, Yulong Chen, Bin Liu

**Affiliations:** Department of Breast Surgery, The Second Hospital of Jilin University, Changchun, China

**Keywords:** breast cancer, tumor-stroma ratio, survival, triple-negative breast cancer, meta-analysis

## Abstract

**Background:**

Tumor-stroma ratio (TSR) has been suggested as an emerging prognostic predictor in women with breast cancer. However, previous studies evaluating the association between TSR and survival in women with breast cancer showed inconsistent results. We performed a meta-analysis to systematically evaluate the possible prognostic role of TSR in breast cancer.

**Methods:**

Relevant cohort studies were obtained *via* search of PubMed, Embase, and Web of Science databases. A random-effects model, which incorporated the potential heterogeneity, was used to pool the results.

**Results:**

Twelve cohort studies with 6175 patients were included. Nine of the 12 studies used 50% as the cutoff to divide the patients into those with stroma-rich (low TSR) and stroma-poor (high TSR) tumors. Pooled results showed that compared women with stroma-poor tumor, those with stroma-rich tumor were associated with worse survival outcomes (disease-free survival [DFS]: hazard ratio [HR] = 1.56, 95% confidence interval [CI]: 1.32 to 1.85, P < 0.001; overall survival [OS]: HR = 1.67, 95% CI: 1.46 to 1.91, P < 0.001; and cancer-specific survival [CSS]: HR = 1.75, 95% CI: 1.40 to 2.20, P < 0.001). Analysis limited to women with triple-negative breast cancer (TNBC) showed consistent results (DFS: HR: 2.07, 95% CI: 1.59 to 2.71, P < 0.001; OS: HR: 2.04, 95% CI: 1.52 to 2.73, P < 0.001; and CSS: HR: 2.40, 95% CI: 1.52 to 3.78, P < 0.001).

**Conclusions:**

Current evidence from retrospective studies supports that tumor TSR is a prognostic predictor or poor survival in women with breast cancer.

## Introduction

Breast cancer remains one of the most common malignancies in women (1, 2). Currently, breast cancer is mainly classified by the presence or absence of molecular markers (3). Alterations in the tumor microenvironment have recently been recognized as a major participant in the progression of the disease (4, 5). Tumor stroma, which refers to a complex mixture of non-neoplastic cells, involving endothelial cells, fibroblasts, and immune cells embedded in the extracellular protein matrix, has been confirmed to a key role in the carcinogenesis and metastasis (6). Subsequently, the tumor-stroma ratio (TSR), which represents the amount of tumor-associated stroma at invasive tumor on traditional hematoxylin and eosin (H&E)-stained paraffin sections, has been proposed to be a predictor of poor prognosis in solid tumor (7). Indeed, a previous meta-analysis showed that high proportion of stroma in cancer tissue was associated with poor clinical outcomes, although studies with various types of cancer were included and a site-specific association between TSR and survival in patients with solid tumor was suggested (8). Some studies have been performed to evaluate the association between TSR and survival outcomes in women with breast cancer (9–20), but the results were not always consistent. Women with stroma-rich breast cancer were shown to have poor survival in some studies (9–13, 15–17, 19, 20), but not in others (14, 18). Therefore, we performed a meta-analysis to evaluate the association between TSR and survival in women with breast cancer. Particularly, since triple-negative breast cancer (TNBC) is an aggressive form of breast cancer without the expressions of hormonal receptors (21), we also evaluated the potential prognostic role of TSR in women with TNBC.

## Methods

The meta-analysis was performed in accordance with the MOOSE (Meta-analysis of Observational Studies in Epidemiology) (22) and Cochrane’s Handbook (23) guidelines.

### Literature Search

Studies were identified *via* systematic search of electronic databases of PubMed, Embase, and Web of Science *via* the following terms: (1) “tumor-stroma” OR “tumour-stroma” OR “tumor stroma” OR “tumour stroma” OR “Glasgow tumor microenvironment score”; (2) “breast cancer”; and (3) “prognosis” OR “survival” OR “recurrence” OR “deaths” OR “outcome” OR “mortality”. The search was limited to human studies published in English or Chinese. The reference lists of related original and review articles were also analyzed using a manual approach. The final literature search was performed on May 3, 2021.

### Study Selection

The inclusion criteria for the studies were: (1) cohort studies; (2) included women with confirmed diagnosis of primary breast cancer; (3) evaluated the association between TSR and survival outcomes of the patients, including disease-free survival (DFS), overall survival (OS), and cancer-specific survival (CSS); (4) reported the hazard ratio (HR) for at least one of the above survival outcomes comparing between women with stroma-rich (low TSR) and stroma-poor (high TSR) breast cancer; and (5) multivariate analysis was used for determine HR, at least after adjustment of age of the women. Reviews, editorials, preclinical studies, and studies irrelevant to the aim of current meta-analysis were excluded.

### Data Extracting and Quality Evaluation

Literature search, data extraction, and quality assessment of the included studies were independently performed by two authors according to the predefined criteria. Discrepancies were resolved by consensus or discussion with the corresponding author. The extracted data included: (1) name of first author, publication year, and country where the study was performed; (2) study design characteristics; (3) patient characteristics, including diagnosis of the women, sample size, and duration of enrollment; (4) cutoff values for TSR; (5) outcomes reported; and (6) confounding factors that were included in the multivariate analyses. The quality of each study was evaluated using the Newcastle-Ottawa Scale (24) which ranges from 1 to 9 stars and judges each study regarding three aspects: selection of the study groups; the comparability of the groups; and the ascertainment of the outcome of interest.

### Statistical Analyses

We used HRs and their corresponding 95% confidence intervals (CIs) as the general measure for the prognostic efficacy of TSR for survival in women with breast cancer. Data of HRs and their corresponding standard errors (SEs) were calculated from 95% CIs or p values, and were logarithmically transformed to stabilize variance and normalized the distribution (23). The Cochrane’s Q test and estimation of I^2^ statistic were used to evaluate the heterogeneity among the include cohort studies (25). A significant heterogeneity was considered if I^2^ > 50%. We used a random-effects model to synthesize the RR data because this model is considered as a more generalized method which incorporates the potential heterogeneity among the included studies (23). Sensitivity analyses, by omitting one individual study at a time, were performed to test the robustness of the results (26). Subgroup analyses limited to women with TNBC were further performed. The potential publication bias was assessed by funnel plots with the Egger’s regression asymmetry test (27). A P value < 0.05 indicates statistically significance. We used the RevMan (Version 5.1; Cochrane Collaboration, Oxford, UK) and Stata software for the meta-analysis and statistics.

## Results

### Literature Search

**Figure 1:**
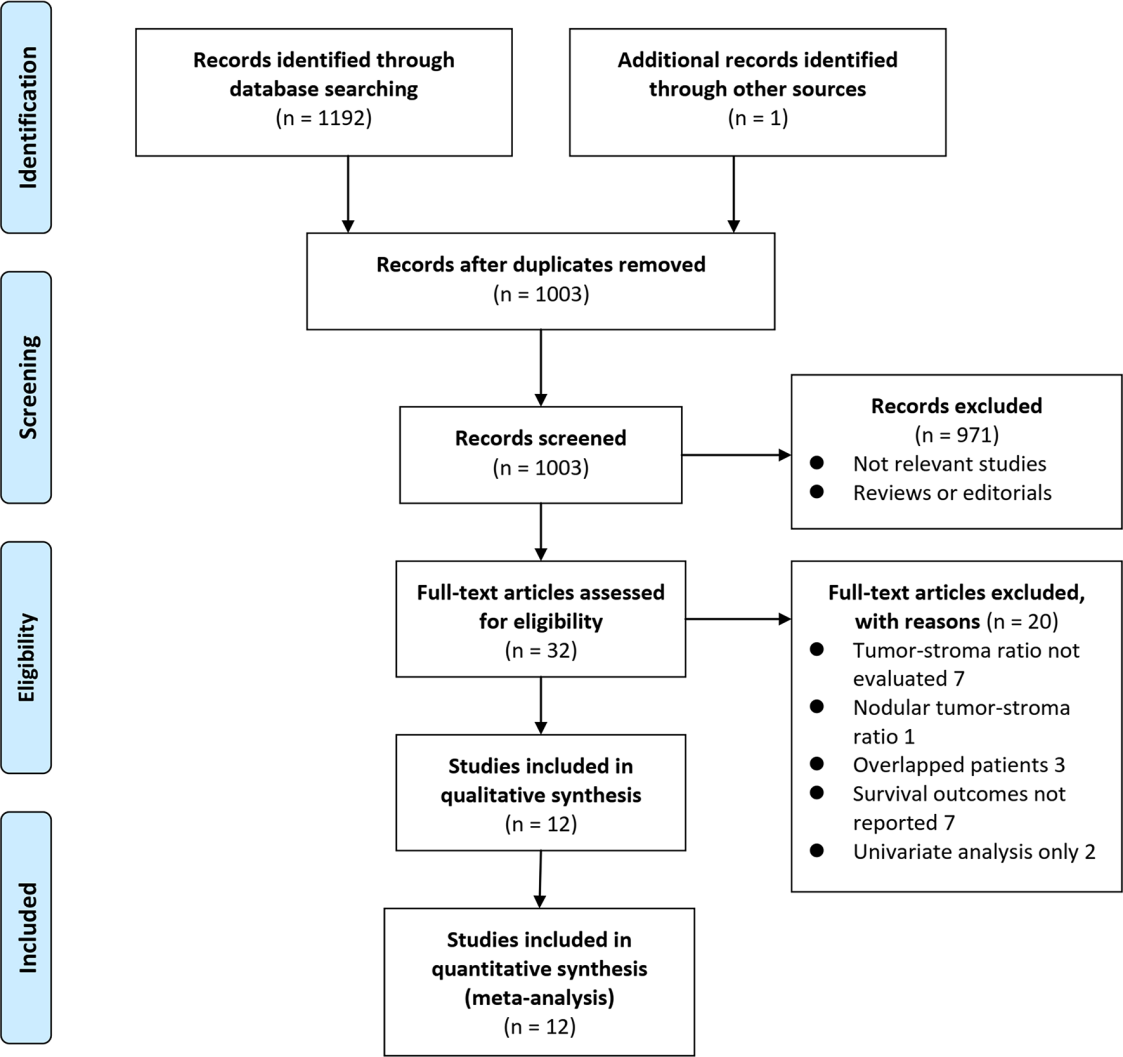
Flowchart of literature search.

The process of database search was summarized in **Figure 1**. Briefly, 1193 articles were identified by initial literature search of PubMed, Embase, and Web of Science databases (n = 1192) and by screening of the references of related reviews and studies (n = 1). Among them, 1003 articles remained after excluding of duplications and 971 were excluded through screening of the titles and abstracts mainly because they were not relevant to the purpose of the meta-analysis. Subsequently, 32 potential relevant records underwent full-text review. Of these, 20 were further excluded based on reasons listed in **Figure 1**. Finally, twelve studies were included (9–20).

### Study Characteristics and Quality Evaluation

The characteristics of the included studies were summarized in **Table 1**. All of the included studies were retrospective cohort including women diagnosed with BC from the Netherlands, United Kingdom, Australia, Sweden, and Ukraine. The sample size for the studies varied between 45 and 1794. In nine of the twelve cohort studies, 50% was set as the cutoff for TSR (9–13, 15, 17, 18, 20), while in the remaining three studies, medians (16, 19) or optimal cutoff calculated by a log-rank test were used (14). Outcome of DFS was reported in eight studies (9–11, 14, 15, 17, 18, 20), while OS and CSS were reported in ten (9–12, 14–16, 18–20) and three (13, 16, 17) studies, respectively. Potential confounding factors, such as age, histological type, tumor size, stage, grade, and the status of hormonal receptors, were adjusted in the multivariate analyses in the original studies. The NOS scores of the included studies ranged from seven to nine, indicating generally good study quality (**Table 2**).

**Table 1:** Characteristics of the included cohort studies.

Study	Country	Design	Patient characteristics	Sample size	Duration	Cutoff for TSR	Outcomes reported	Variables adjusted	NOS
de Kruijf et al., 2011 (9)	the Netherlands	RC	Women with early BC primarily treated with surgery	574	1985~1994	50%	DFS, OS	Age, grade, histological type, tumor stage, ER/HR/PR status, Ki67, chemotherapy, and endocrine therapy	8
Moorman et al., 2012 (10)	the Netherlands	RC	Women with TNBC who underwent surgery	124	2004~2008	50%	DFS, OS	Age, family history of BC, multifocality, tumor stage, lymphovascular invasion, and mitotic activity index	9
Dekker et al., 2013 (11)	the Netherlands	RC	Women with early BC who were not previously treated	403	NR	50%	DFS, OS	Age, tumor stage, tumor grade, Ki67, perioperative chemotherapy, p53 expression, and the type of procedure	8
Downey et al., 2014 (12)	UK	RC	Women with ER-positive BC	118	1994~1997	50%	OS	Age, tumor size, grade and lymph node status	8
Gujam et al., 2014 (13)	UK	RC	Women with primary operable invasive ductal BC	361	1995~1998	50%	CSS	Age, tumor size, grade, lymph node status, ER/HR/PR status, T-lymphocyte infiltrate, and loco-regional and systemic adjuvant therapy	8
Downey et al., 2015 (14)	UK	RC	Women with inflammatory BC	45	2005~2013	46% for DFS, 31% for OS	DFS, OS	Age, tumor size, grade, and ER/HR/PR status	7
Roeke et al., 2017 (15)	the Netherlands	RC	Women with primary operable BC	737	1990~1999	50%	DFS, OS	Age, tumor size, tumor grade, nodal status, subtype, ER/HR/PR status, and adjuvant therapy	9
Vangangelt et al., 2020a (17)	the Netherlands	RC	Women with BC aged 70 years and older	619	1997~2004	50%	DFS, OS	Age, tumor size, tumor grade, histological type, and ER/HR/PR status	8
Millar eta l., 2020 (16)	Australia	RC	Women with luminal and triple negative BC	647	1996~2003	Median	CSS, OS	Age, size, grade, T-lymphocyte infiltrate, lymph nodal status and chemotherapy	8
Vangangelt et al., 2020b (18)	UK	RC	Women with BC treated primarily with surgery	1,794	1993~2002	50%	DFS, OS	Age, tumor grade, size, histological type, ER/HR/PR status	9
Micke et al., 2021 (19)	Sweden	RC	Women with BC treated with surgery	521	1987~2004	Median	OS	Age, tumor grade, size, stage, and performance status	8
Zakhartseva and Yanovytska, 2021 (20)	Ukraine	RC	Women with primary stage I-III TNBC	232	2009~2018	50%	DFS, OS	Age, tumor grade, size, and histological type	8

TSR, tumor-stroma ratio; NOS, Newcastle-Ottawa Scale; UK, United Kingdom; RC, retrospective cohort; BC, breast cancer; TNBC, triple-negative breast cancer; NR, not reported; DFS, disease-free survival; OS, overall survival; CSS, cancer-specific survival; ER, estrogen receptor; PR, progesterone receptor; human epidermal growth factor receptor 2.

**Table 2:** Details of study quality evaluation *via* the Newcastle-Ottawa Scale.

Study	Representativeness of the exposed cohort	Selection of the non-exposed cohort	Ascertainment of exposure	Outcome not present at baseline	Control for age	Control for other confounding factors	Assessment of outcome	Enough long follow-up duration	Adequacy of follow-up of cohorts	Total
de Kruijf et al., 2011 (9)	0	1	1	1	1	1	1	1	1	8
Moorman et al., 2012 (10)	1	1	1	1	1	1	1	1	1	9
Dekker et al., 2013 (11)	0	1	1	1	1	1	1	1	1	8
Downey et al., 2014 (12)	0	1	1	1	1	1	1	1	1	8
Gujam et al., 2014 (13)	0	1	1	1	1	1	1	1	1	8
Downey et al., 2015 (14)	0	1	1	1	1	1	1	1	0	7
Roeke et al., 2017 (15)	1	1	1	1	1	1	1	1	1	9
Vangangelt et al., 2020a (17)	0	1	1	1	1	1	1	1	1	8
Millar et al., 2020 (16)	0	1	1	1	1	1	1	1	1	8
Vangangelt et al., 2020 (18)	1	1	1	1	1	1	1	1	1	9
Micke et al., 2021 (19)	0	1	1	1	1	1	1	1	1	8
Zakhartseva and Yanovytska, 2021 (20)	0	1	1	1	1	1	1	1	1	8

### TSR and Survival in Breast Cancer

**Figure 2:**
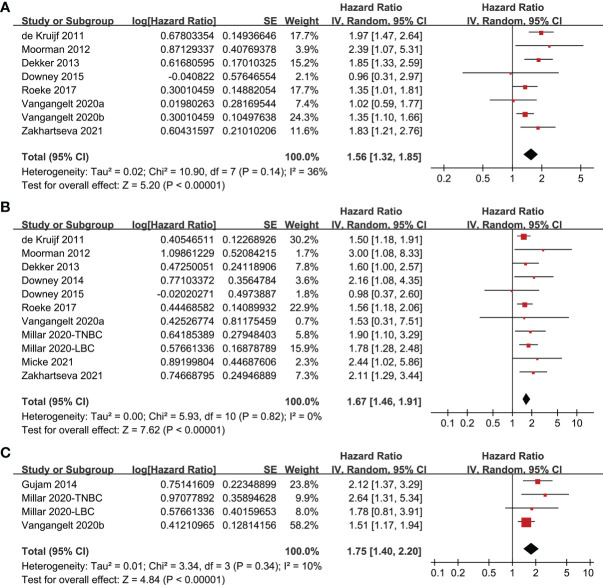
Forest plots for the meta-analysis of the association between TSR and survival in women with breast cancer; **(A)**, association between TSR and DFS in women with breast cancer; **(B)**, association between TSR and OS in women with breast cancer; and **(C)**, association between TSR and CSS in women with breast cancer.

Since one study reported data of women with triple-negative and luminal breast cancer separately, these two datasets were indepdently included into the meta-analysis (16). Pooled results of eight studies (9–11, 14, 15, 17, 18, 20) showed that compared to women with stroma-poor breast cancer, women with stroma-rich tumor were associated with poor DFS (HR = 1.56, 95% CI: 1.32 to 1.85, P < 0.001; **Figure 2A**) with moderate heterogeneity (I^2^ = 36%). Sensitivity analyses by excluding one study at a time showed consistent results (HR: 1.46~1.65, P all < 0.05). In addition, pooling the results of 11 datasets from ten studies (9–12, 14–16, 18–20) indicated that women with stroma-rich tumor were associated with poor OS (HR: 1.67, 95% CI: 1.46 to 1.91, P < 0.001; **Figure 2B**) with no significant heterogeneity (I^2^ = 0%). Sensitivity analyses by excluding one study at a time did not significantly change the results (HR: 1.64~1.75, P all < 0.05). Moreover, meta-analysis of four datasets from three studies (13, 16, 17) showed that women with stroma-rich tumor had poor cancer-specific survival (HR = 1.75, 95% CI: 1.40 to 2.20, P < 0.001; **Figure 2C**) with mild heterogeneity (I^2^ = 10%). Also, sensitivity analyses by excluding one dataset at a time did not significantly change the results (HR: 1.65~2.16, P all < 0.05).

### TSR and Survival in TNBC

**Figure 3:**
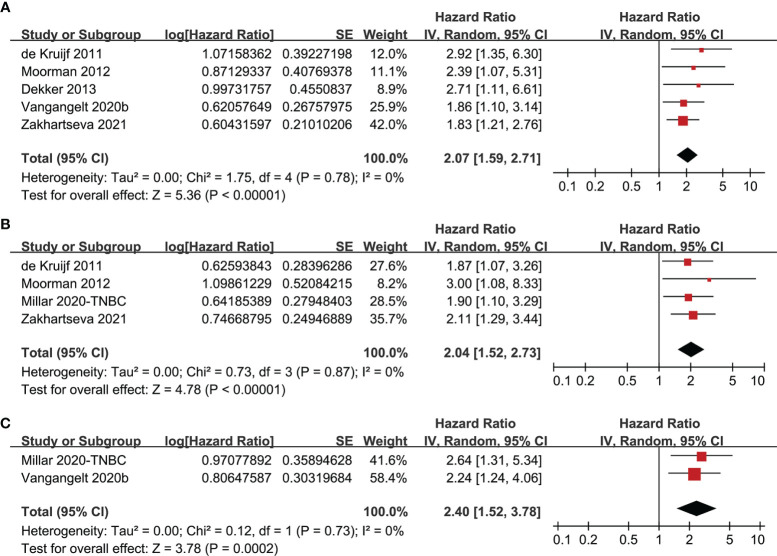
Forest plots for the meta-analysis of the association between TSR and survival in women with TNBC; **(A)**, association between TSR and DFS in women with TNBC; **(B)**, association between TSR and OS in women with TNBC; and **(C)**, association between TSR and CSS in women with TNBC.

Further meta-analyses limited in studies of TNBC also showed that women with stroma-rich tumor were associated with worse survival outcomes, including DFS (five studies, HR: 2.07, 95% CI: 1.59 to 2.71, P < 0.001; I^2^ = 0%; **Figure 3A**), OS (four studies, HR: 2.04, 95% CI: 1.52 to 2.73, P < 0.001; I^2^ = 0%; **Figure 3B**), and CSS (two studies, HR: 2.40, 95% CI: 1.52 to 3.78, P < 0.001; I^2^ = 0%; **Figure 3C**).

### Publication Bias

**Figure 4:**
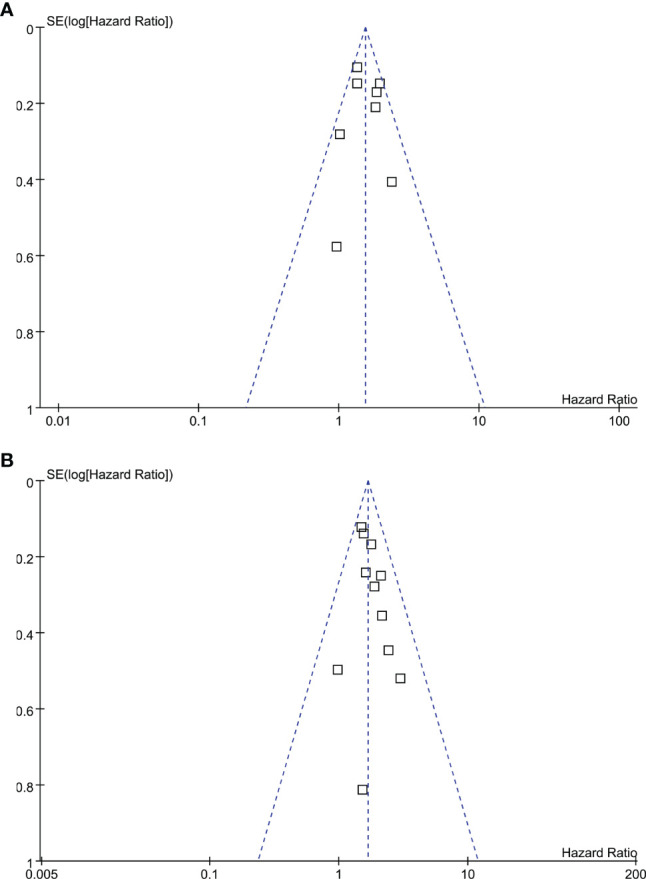
Funnel plots for the publication bias of the meta-analysis of the association between TSR and survival in women with breast cancer; **(A)**, DFS; and **(B)**, OS.

The funnel plots for the meta-analysis of the association between TSR with DFS and OS ere shown in **Figures 4A, B**. The plots were symmetrical on visual inspection, suggesting low risks of publication biases. Results of Egger’s regression tests also suggested low risks of publication biases (P = 0.358 and 0.169, respectively). Publication biases for the other meta-analyses were difficult to estimate since limited available datasets were included.

## Discussion

In this meta-analysis, by combining the results of available cohort studies, we found that women with stroma-rich (low TSR) breast cancer were associated with significantly worse survival as compared to those with stroma-poor (high TSR) tumor. Results of sensitivity analysis showed that the results of the meta-analysis were not primarily driven by either of the included studies, indicating the robustness of the finding. Further analysis limited to studies including TNBC only showed that TSR in primary tumor remains an independent prognostic predictor of poor survival in these women. Taken together, current evidence from retrospective studies supports that tumor TSR is a prognostic predictor or poor survival in women with breast cancer.

To the best of our knowledge, this is the first meta-analysis evaluating the association between TSR in primary tumor and survival outcomes in women with breast cancer. The strengths of the meta-analysis include the followings. Firstly, extensive literature retrieval and strict inclusion criteria were applied. Accordingly, the up-to-date of literatures regarding the prognostic role of TSR in breast cancer were retrieved. In addition, only studies with multivariate analysis were included. Variables such as age, histological type, tumor size, stage, grade, and the status of hormonal receptors, were adjusted when the HR for the association between TSR and survival of breast cancer was estimated. Therefore, results of the meta-analysis indicated that the association between TSR and survival in these patients may be independent of the above confounding factors. Finally, the stability of the finding was validated in the consistent results of different survival outcomes (DFS, OS, and CSS), of the “leave-one-out” sensitivity analyses, and of the additional analyses limited to women with TNBC only.

The mechanisms underlying the potential role of components of tumor stoma in the progression of breast cancer are multifactorial (28). For example, cancer-associated fibroblasts (CAFs), as a major component of cancer stroma, could promote tumor proliferation, invasion and metastasis and induce angiogenesis *via* the production and secretion of various cytokines and growth factors (29). Besides, remodeling of the extracellular matrix (ECM) by degrading proteases is also shown to be involved in the metastasis of breast cancer (30). Theoretically, it has been recognized that tumor microenvironment, such as stroma of tumor plays important role in the pathogenesis and progression of breast cancer (31). However, index of tumor microenvironment or stroma has not been integrated in the risk stratification and determination of treatments for women with breast cancer. Besides, TSR could be obtained by conventional pathological analysis with a microscope, which is simple, inexpensive, effective, and feasible in real-world clinical practice. Moreover, treatments targeting tumor stroma such as CAFs and the components of ECM may be effective and promising (32, 33). Once therapy targeting tumor stroma becomes critical, evaluation of TSR in women with breast cancer may be helpful to identify patients with optimal therapy response. Taken together, although these results should be validated in large-scale prospective cohort studies, results of the meta-analysis suggested that TSR may be an independent prognostic predictor for the survival of breast cancer. These findings support the incorporation of TSR into the risk stratification for women with breast cancer.

Our study has limitations. Firstly, studies available for the meta-analysis were retrospective, which may be confounded by the recall or selection biases. Therefore, prospective cohort studies are needed for validation. Secondly, the optimal cutoff of TSR for defining of stroma-rich and stroma-poor breast cancer remains to be determined. In addition, since this is a meta-analysis based on data of study level, we were unable to determine whether the prognostic role of TSR on survival of breast cancer could be affected by patient or tumor characteristics, such as age, ethnicity, and comorbidities of the women, histological and molecular type of the tumor, and concurrent anticancer treatments. In particular, since the associations between TSR and survival outcomes according to the other molecular subtypes of breast cancer were rarely reported in the included studies, we were unable to determine this in our meta-analysis. Large-scale prospective cohort studies and future meta-analysis based on individual-patient data may be considered for further evaluation. Finally, although the methods for TSR analysis among the included studies were standard, personal subjectivity during the process of TSR analysis could still affect the results.

In conclusion, results of the meta-analysis showed that women with stroma-rich breast cancer defined by low TSR are associated with worse survival as compared to those with stroma-poor cancer. Although large-scale prospective cohort studies are needed for validation, results of the meta-analysis suggested that TSR may be an independent prognostic predictor of the survival in women with breast cancer.

## Data Availability Statement

The original contributions presented in the study are included in the article/supplementary material. Further inquiries can be directed to the corresponding author.

## Author Contributions

PJ and BL conceived and designed the study. PJ and YC selected the studies and collected the data. PJ and BL analyzed data. All authors interpreted the results. PJ drafted the paper. All authors revised the draft paper. All authors contributed to the article and approved the submitted version.

## Funding

This study was supported by the grants from Jilin Provincial Science and Technology Development Project (#20200201577JC).

## Conflict of Interest

The authors declare that the research was conducted in the absence of any commercial or financial relationships that could be construed as a potential conflict of interest.

## Publisher’s Note

All claims expressed in this article are solely those of the authors and do not necessarily represent those of their affiliated organizations, or those of the publisher, the editors and the reviewers. Any product that may be evaluated in this article, or claim that may be made by its manufacturer, is not guaranteed or endorsed by the publisher.
